# Supplementary material for: Excess centrosomes disrupt vascular lumenization and endothelial cell adherens junctions
Source: Angiogenesis. 2020 Jul 22;23(4):567–75. doi: 10.1007/s10456-020-09737-7 (PMC7524686; doi:10.1007/s10456-020-09737-7)
Supplement: Supplementary file 1 — (PDF 3020 kb) [file 10456_2020_9737_MOESM1_ESM.pdf]

SUPPLEMENTARY MATERIAL

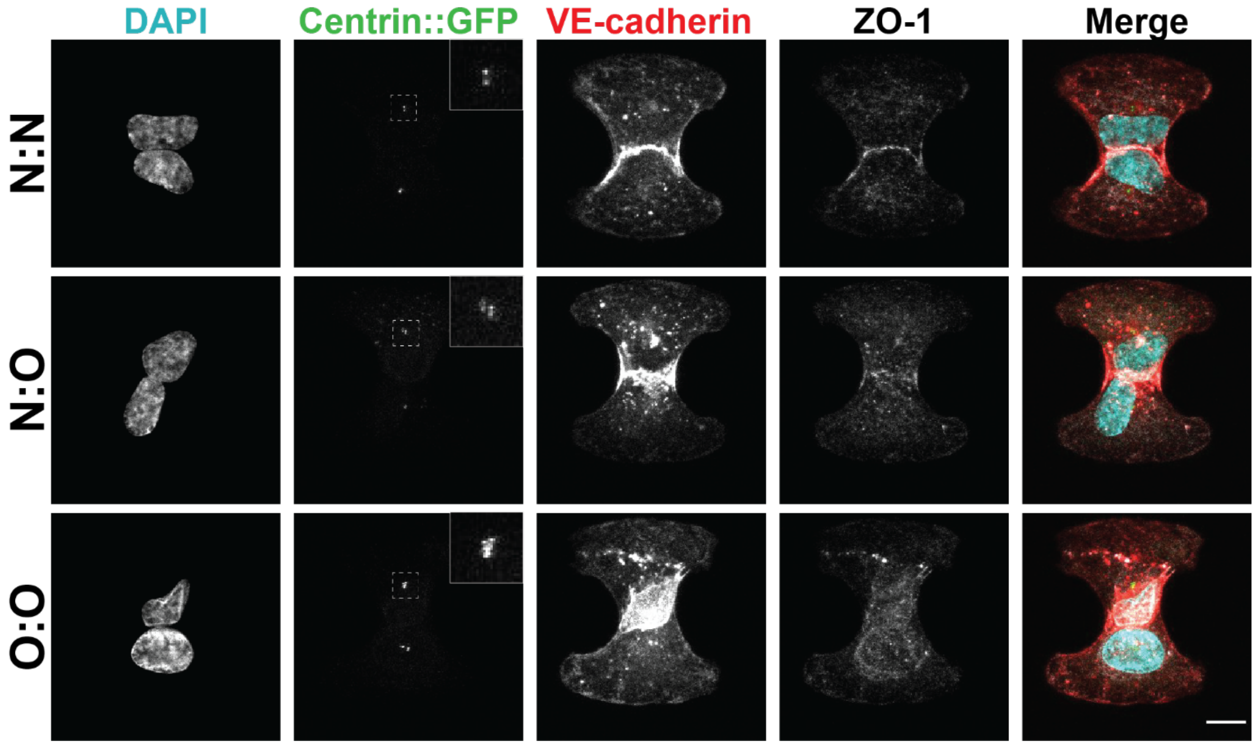

Supplementary Figure 1. EC with excess centrosomes do not form proper tight junctions.

Representative images of micropatterns with N:N, N:O, or O:O configurations. EC were labeled for DNA (cyan, DAPI), centrosomes (green, centrin::GFP), adherens junctions (VE-cadherin, red), and tight juntions (ZO-1, gray). Insets, centrin-GFP. Scale bar, 10μm.

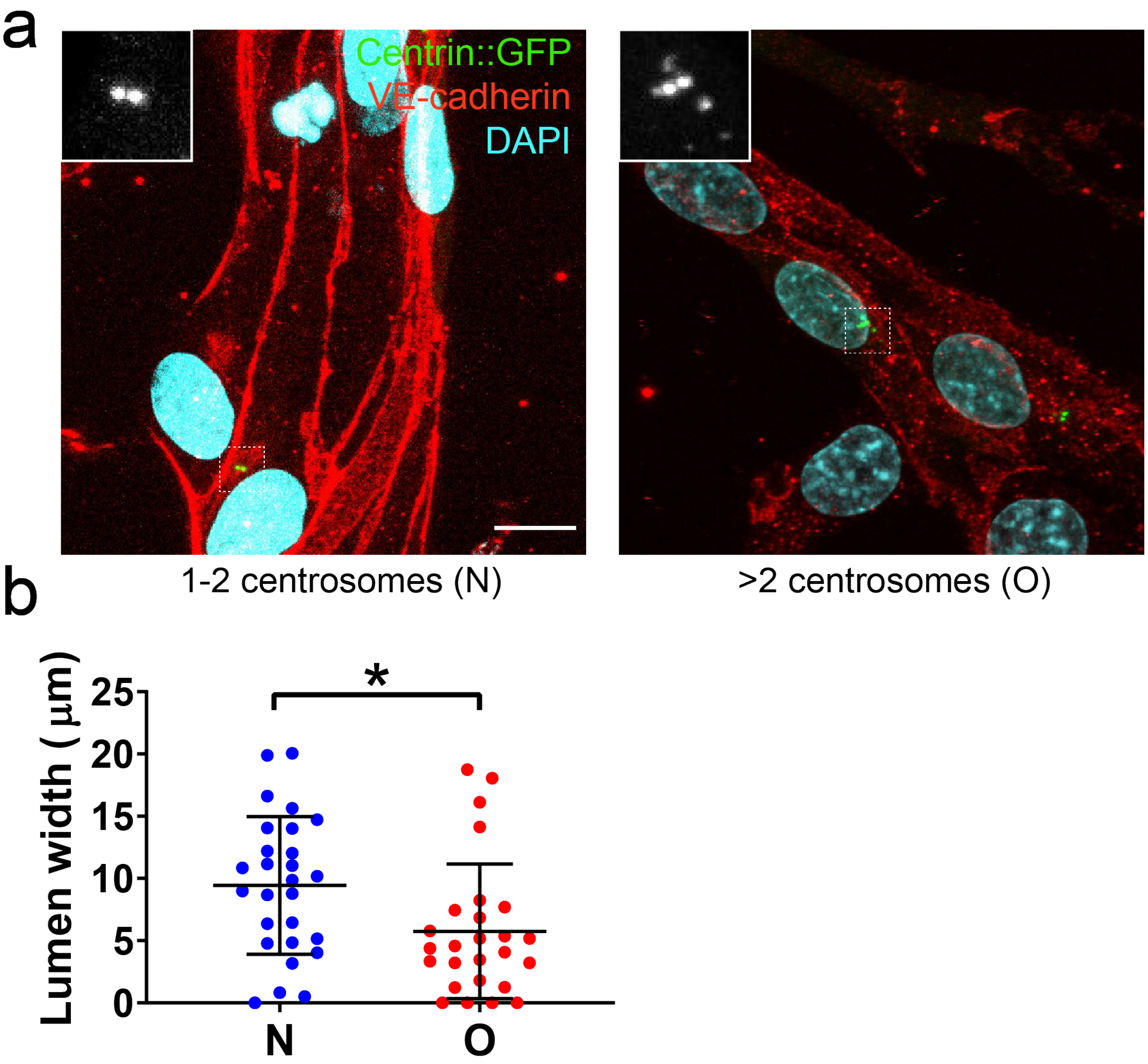

Supplementary Figure 2. EC with excess centrosomes have disrupted junctions and lumens in 3D sprouts.

(a) Representative images of 3D sprouts showing junctions in EC with indicated centrosome number. EC are labeled for DNA (cyan, DAPI), centrosomes (green, centrin::GFP), and adherens junctions (VE-cadherin, red). Insets, centrin-GFP. Scale bar, 10 μm. **(b)** Quantification of raw lumen width (not normalized to vessel width). n=3 replicates. Statistics: one-way ANOVA with Tukey's correction.

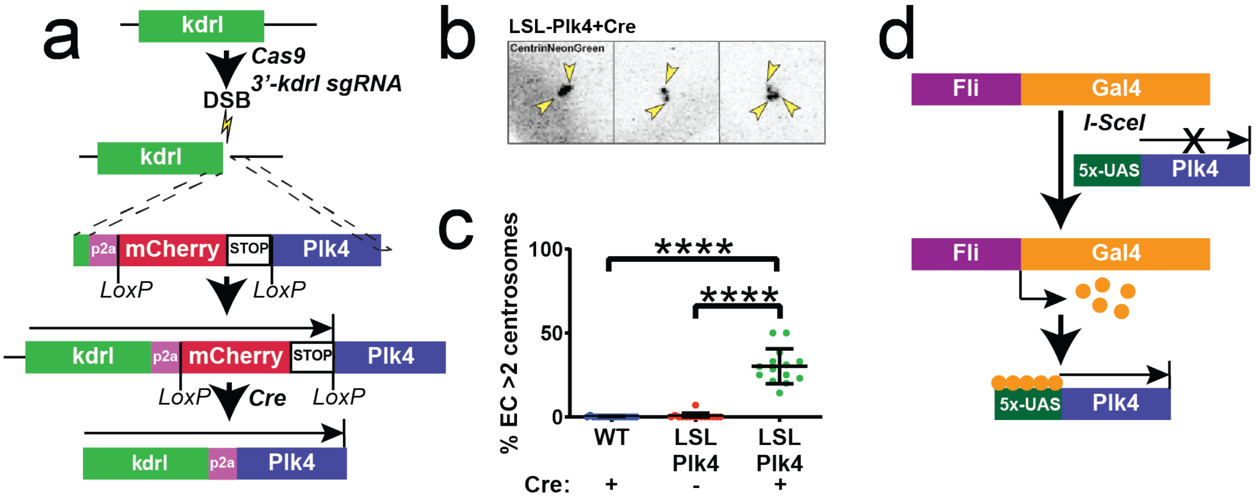

Supplementary Figure 3. *Plk4* overexpression in zebrafish leads to excess centrosomes.

(a) Schematic for Cre-mediated *Plk4* overexpression in zebrafish. **(b)** Representative image of *Plk4* overexpression in 72 hpf zebrafish. Yellow arrowheads, EC centrosome number. **(c)** Quantification of EC with >2 centrosomes in zebrafish with indicated genotypes/conditions (WT + Cre, n=17 fish; LSL-Plk4 uninjected, n=15; LSL-Plk4 + Cre, n=14). Statistics: one-way ANOVA with Tukey's correction. ****, $p \leq 0.0001$. **(d)** Schematic for transient *Gal4-UAS* mediated overexpression of *Plk4* in zebrafish.
